# Troxerutin and Cerebroprotein Hydrolysate Injection Protects Neurovascular Units from Oxygen-Glucose Deprivation and Reoxygenation-Induced Injury* In Vitro*

**DOI:** 10.1155/2018/9859672

**Published:** 2018-05-02

**Authors:** Hóngyi Zhào, Yu Liu, Jing Zeng, Dandan Li, Weiwei Zhang, Yonghua Huang

**Affiliations:** ^1^Department of Neurology, PLA Army General Hospital, 5 Nanmencang Dongsishitiao Street, Beijing 100007, China; ^2^Department of Neurology, No. 261 Hospital of PLA, 116 Zaojiatun Shangzhuang, Beijing 100094, China

## Abstract

Cerebral ischemia/reperfusion (I/R) injury involves complex events of cellular and molecular processes. Previous studies suggest that a neurovascular unit (NVU) acts as an intricate network to maintain the neuronal homeostatic microenvironment. The present study established an NVU model for oxygen-glucose deprivation and reoxygenation (OGD/R) damage, trying to target the major components of the NVU using a coculture of rat neurons, astrocytes, and rat brain microvascular endothelial cells (rBMECs) to investigate the therapeutic effects of troxerutin and cerebroprotein hydrolysate injections (TCHis). The study observed that OGD/R downregulated the expressions of GAP-43, Claudin-5, and AQP-4 obviously detected by Western blotting and immunocytochemical analysis, respectively, while TCHi ameliorated the effect of OGD/R significantly. Meanwhile, TCHi alleviated the abnormalities of ultrastructure of neurons and rBMECs induced by OGD/R. Furthermore, both levels of inflammatory cytokines (IL-1*β*, IL-6, and TNF-*α*) and cell adhesion molecules (VCAM-1 and ICAM-1) detected by ELISA in NVU supernatant were found elevated significantly through OGD/R, but TCHi ameliorated the trend. In addition, TCHi also mitigated the increase of proapoptotic factors (Bax, p53, and caspase-3) induced by OGD/R in NVU model statistically. All these findings demonstrated that TCHis played a protective role, which was reflected in anti-inflammation, antiapoptosis, and blood–brain barrier maintenance. The results of the study concluded that the NVU is an ideal target and TCHi acts as a neuroprotective agent against cerebral I/R injuries.

## 1. Introduction

Cerebral ischemia/reperfusion (I/R) injury is a complex pathological process in the nervous system, resulting in high disability and mortality worldwide, with significant clinical and socioeconomic impacts [1]. The complex pathobiological mechanisms of this medical problem include inflammation, apoptosis, oxidative damage, and ionic imbalances [2]. Although reperfusion after ischemia is essential for cell survival, it may have numerous negative consequences such as microvascular damage, cell dysfunction, and cell death. It can attract leukocytes and cause the release of several proinflammatory mediators and the induction of microglia and macrophages [3]. Excessive neuroinflammation can increase brain damage and bring about many secondary complications that influence stroke outcome; therefore, anti-inflammation is considered a target for ischemic stroke.

A neurovascular unit (NVU), consisting mainly of microvessels, astrocytes, neurons, extracellular matrix, and other types of gliocytes, is defined as a complete functional and structural unit of the brain [4]. Not only do neurons suffer from strokes, but the microvasculature and gliocytes are also involved [5]. Consequently, protecting different cell types simultaneously in the NVU is necessary for I/R injury therapy [6].

Troxerutin, a naturally occurring flavonoid, is known mainly because of its anti-inflammatory, antioxidative, antithrombotic, antineoplastic, and antiapoptotic activities [7, 8]. Cerebroprotein hydrolysate (with abundant bioactive peptides) was found to facilitate the distribution of troxerutin and had a positive synergistic effect with troxerutin against acute ischemic stroke [9]. The present study aimed to investigate the protective effects of troxerutin and cerebroprotein hydrolysate injections (TCHis) on oxygen-glucose deprivation and reoxygenation- (OGD/R-) inducing NVU dysfunction and the possible mechanism.

## 2. Methods

### 2.1. Animals

Adult Wistar rats (3 months old) were purchased from Peking Vital River Laboratory Animal Ltd. Three female rats were mated with one male rat in each cage, and the pregnant females were kept individually. The rat pups were used for further experiments. All experiments were performed in accordance with China's Guidelines for Care and Use of Laboratory Animals.

### 2.2. Primary Cell Cultures

Primary cells were extracted from the rat pups and routinely cultured in conditioned incubators (37°C/5% CO_2_). The isolation procedure was performed according to the methods used by Xue et al. (2013) [10] and Wang et al. (2015) [11].

Primary cortical neurons (N) were prepared from Wistar newborn rats (less than 24 h). In brief, the cerebral cortex was digested with 0.125% trypsin for 10 min at 37°C and the cell suspension was passed through a 75 *μ*m pore filter. Cells were harvested and seeded on poly-D-lysine (Sigma Aldrich, MO, USA) precoated plates in Neurobasal Medium (Invitrogen, CA, US) containing 2% B27 supplement (Invitrogen), 1% penicillin–streptomycin, and 2 mM L-glutamine. Experiments were performed for 8 days* in vitro*.

Primary astrocytes (A) were extracted from 1- to 2-day-old rat pups, as described previously with a few modifications (Saini MG et al., 2011). In brief, the cerebral cortex was digested with 0.125% trypsin for 10 min at 37°C, and the cell suspension was then passed through a 75 *μ*m pore filter. Cells were seeded in the DMEM/F12 medium containing 10% fetal bovine serum (FBS) (NQBB, Australia) and 1% penicillin–streptomycin. After 7–10 days, the cultures were shaken at 37°C at a speed of 260 rpm for 16 h to remove contaminating microglia and oligodendrocytes. The third passage of astrocytes was used for the following study.

Primary brain microvascular endothelial cell (rBMEC) cultures were established from 7-day-old rat pup brain tissues (B), which were extracted and homogenized with type II collagenase/DNaseI (Sigma) for 1 h. Microvessels were separated after density centrifugation (spun at 1000 g/min for 20 min) in 20% bovine serum albumin at 4°C. The microvessels were then digested using a collagenase/dispase solution (Roche Applied Science, Mannheim, Germany) containing DNaseI for 1 h and suspended in a DMEM high-glucose medium containing 20% FBS, 10 ng/mL basic fibroblast growth factor, 30 U/mL heparin, 2 mM glutamine, and 1% penicillin–streptomycin. Cells were then seeded into gelatin (1%) coated flasks, and the third passage of rBMECs was used in this study.

### 2.3. Establishment of NVU* In Vitro*

**Figure 1:**
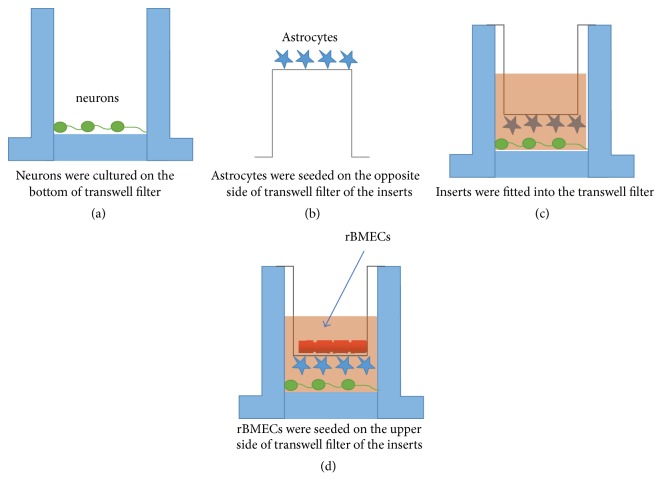
*In vitro* NVU model. Sequential steps are shown in (a), (b), (c), and (d).

**Figure 2:**
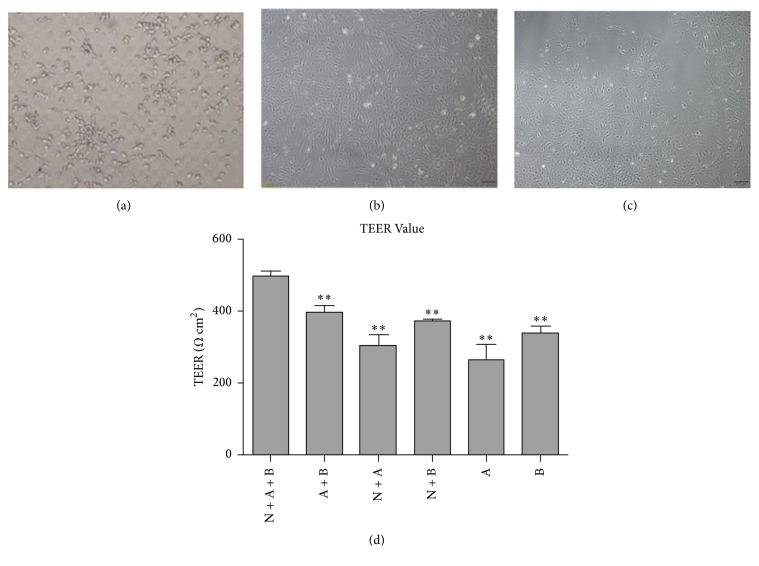
Morphologies of the three types of cells in NVU models. (a), (b), and (c) are neurons, rBMECs, and astrocytes in visible light under the fluorescent inverted microscope, respectively. (d) reveals the TEER values of different culture models, which indicate that the BBB of the NVU model is intact.

The NVU model was established according to the previous report using purified normal morphological cells (Figure 1) [12]. Briefly, after the neurons had grown in a six-well culture plate with a density of 0.5 × 10^5^ cells/cm^2^ for 2 days, the purified astrocytes with 1.5 × 10^5^ cells/cm^2^ were seeded on the outer side of the insert membrane, which faced the bottom of the well. After 4 h for astrocyte adhering, the insert was placed into the well with neurons. Two days later, rBMECs (1.0 × 10^5^ cells/cm^2^) were seeded in the inner side of the insert membrane. After being cocultured for 3–5 days, the NVU model was prepared for the following experiments (Figures 1 and 2).

### 2.4. Four-Hour Leakage Detection

Blood–brain barrier (BBB) permeability was evaluated by performing a 4-hour leakage experiment. After 3 days of coculture, the upper inserts were filled with the medium, while the level of the medium in the plates was maintained 0.5 cm lower than the level of the medium in the upper inserts. Inserts with no cells were used as a control. After 4 h, changes in the level of the medium in the top inserts were observed (Figure 2).

### 2.5. Establishment of OGD/R Damaging NVU and TCHi Treatment

The prepared NVU cells were cultured in a conditional medium [glucose-free, 98.5 mM NaCl, 10.0 mM KCl, 1.2 mM MgSO_4_, 0.9 mM Na_2_HPO_4_, 6.0 mM NaHCO_3_, 1.8 mM CaCl_2_, 40 mM sodium lactate, and 20 mM HEPES (Sigma) at a pH of 6.8] and placed in an anaerobic incubator (BINDER CB150, Germany) with conditions of 5% CO_2_, 0.2% O_2_, and 37°C for 2 h (named as Model group). Then, cultures were switched to completely normal conditions with TCHi at concentrations of 10 *μ*M, 100 *μ*M, or 1000 *μ*M for 2 h [TCHi, Jilin Sihuan Pharmaceutical Co. Ltd., was dissolved in sterile phosphate-buffered saline (PBS)]. Cells cultured in media with PBS under normoxic conditions were used as a control (named as CK group).

### 2.6. Transmission Electron Microscopy

The method of using the transmission electron microscope (TEM) was similar to what Liu et al. (2011) [13] and Garbuzova-Davis et al. (2007) [14] mentioned in their study. The cocultures were postfixed in 1% osmium tetroxide (Electron Microscopy Sciences, Inc., PA, USA) in 0.1 M PB for 1 h at room temperature. Following osmication, they were dehydrated in a graded series of acetone dilutions (30%, 50%, 70%, and 95% acetone in water), allowing 10 min for each change. Three 10 min changes in 100% acetone were made, and the cocultures were transferred to a 50 : 50 mix of acetone: LX112 epoxy resin embedding mix (Ladd Research Industries, Burlington, VT). Subsequently, the cocultures were infiltrated with this mix for 1 h under vacuum and were transferred to a 100% LX112 embedding mix and infiltrated for 1 h on a rotator. Two more 1 h infiltration steps were performed with fresh changes of the embedding mixture. Cocultures were further infiltrated in a fresh embedding medium at 4°C overnight. The following day, the tissues were infiltrated in two additional changes of embedding medium at room temperature, 4 h per change, and then embedded in a fresh change of resin in tissue capsules. The blocks were polymerized at 70°C in an oven overnight and then trimmed and sectioned with a diamond knife on a Reichert Ultracut E ultramicrotome (Leica Microsystems, Inc., IL, USA). Thick sections cut at 0.35 m were placed on glass slides and stained with 1% toluidine blue. Thin sections were cut at 80–90 nm, placed on copper grids, and stained with uranyl acetate and lead citrate. The sections were examined and photographed with a Philips CM10 TEM (FEI, Inc., OR, USA) at 60 kV. The arrangement of parenchymal cells and cerebral microvessel endothelial cells on the Transwell filter was detected in the cocultures.

### 2.7. Western Blotting

Cells in the NVU were individually scraped down and lysed on ice for 10 min. After centrifugation (13,000*g*, 4°C), the resulting supernatant was saved as the cytoplasmic extract sample and the nuclear pellet was prepared for a nuclear extract sample. The samples were separated by 10% sodium dodecyl sulfate–polyacrylamide gel electrophoresis (SDS-PAGE) (P0012A, Beyotime, China) and then transferred to polyvinylidene difluoride membranes. The membranes were blocked for 1 h and incubated overnight at 4°C with the following antibodies: rabbit polyclonal antibody against GAP-43 (1 : 1000, 8945S, CST, China), rabbit polyclonal against AQP-4 (1 : 1000, ab31721, Abcam, China), rabbit polyclonal antibody against Claudin-5 (1 : 1000, ABT45, Millipore, China), and mouse polyclonal antibody against Tubulin (1 : 200, sc-5286, Santa Cruz, China). Membranes were incubated with a secondary goat anti-rabbit/mouse antibody (1 : 3,000, Service, China) for 1 h at 37°C. Immunoreactive bands were observed using the ECL detection system (Bio-Rad, Beijing, China).

### 2.8. Immunocytochemical Analysis

For immunocytochemical analysis, cells were blocked with 10% normal goat serum in PBS containing 0.1% Triton X-100 (Sigma) for 30 min before incubation with primary antibodies for 18 h at 4°C: Rabbit polyclonal antibody against Anti-GAP-43 (1 : 100, 8945S, CST, China), rabbit polyclonal against AQP-4 (1 : 100, ab31721, Abcam, China), rabbit polyclonal antibody against claudin-5 (1 : 100, ABT45, Millipore, China), goat monoclonal antibody against caspase-3 (1 : 100, EB07286, Everest Biotech, UK), mouse monoclonal antibody against P53 (1 : 100, ab26, Abcam, China), and rabbit monoclonal antibody against Bax (1 : 250, ab32503, Abcam, China) at 4°C overnight. The sections were subsequently incubated with Sheep Anti-Mouse IgG H&L secondary antibody (Texas Red) (1 : 500, ab6806, Abcam, China), Donkey Anti-Goat IgG H&L (Alexa Fluor 647) (1 : 200, ab150135, Abcam, China), and Alexa Fluor 488 Monkey Anti-Rabbit IgG (H + L) antibody (1 : 1000, A21206, Life Technologies, Beijing, China) at room temperature for 2 h. Subsequently, the sections were incubated with the DAPI dyeing kit (1 : 500, C0060, Solarbio, Beijing, China) for 15 min. The sections were covered with coverslips for microscopic observation after antibody incubation. Images were acquired using a Nikon Eclipse (TE2000-E) inverted C1 confocal microscope (Nikon Instruments, Minato, Tokyo, Japan) equipped with an oil immersion 60x objective with 1.4 numerical aperture (Nikon) or a Zeiss video microscope (Zeiss AG, Oberkochen, Germany) equipped with a plan Neofluar 10x/0.3 numerical aperture. Antibodies of Bax, P53, caspase-3, and DAPI were used for the triple labeling of apoptosis.

### 2.9. Enzyme-Linked Immunosorbent Assay

The contents of the tumor necrosis factor-*α* (TNF-*α*), interleukin-1*β* (IL-1*β*), interleukin-6 (IL-6), vascular cell adhesion molecule 1 (VCAM-1), and intercellular adhesion molecule 1 (ICAM-1) of the supernatant were detected by a sandwich enzyme-linked immunosorbent assay (ELISA) according to the manufacturer's protocol. Absorbances were measured at 495 nm using a microplate ELISA reader (Bio-Rad Model 680 microplate reader, Multiskan FC, Thermo Fisher Scientific Oy, Vantaa, Finland). Each final value was quantified against a standard curve calibrated with known amounts of protein.

### 2.10. Statistical Analysis

All the results were repeated at least three times. All pictures were analyzed by Adobe Photoshop software. Data were statistically analyzed by analysis of variance (using IBM SPSS 17.0). Results were expressed as mean ± SD. *P* < 0.05 was regarded to be statistically significant.

## 3. Result

**Figure 3:**
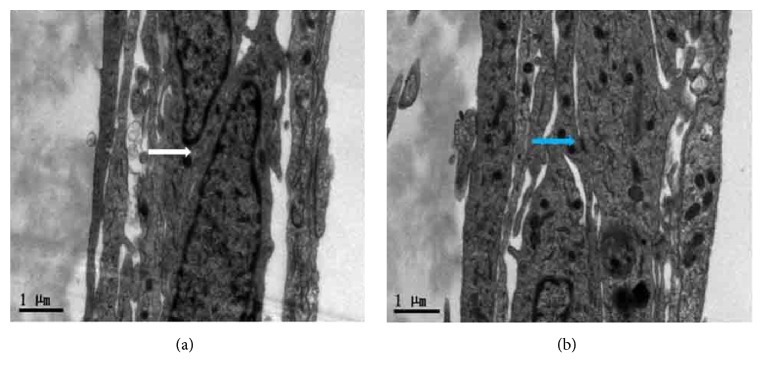
TEM showed that the BBB of rBMECs had intact and continuous tight junctions (white arrow) and desmosomes (blue arrow).

Morphology and specific identification for three types of cells in the coculture system: as shown in Figure 2, pictures of the same field were obtained in visible light under the fluorescent inverted microscope. Neuronal cells were cultured at the bottom of the Transwell filter (Figure 2(a)), rBMECs (Figure 2(b)) were seeded on the upper side of the Transwell filter of the inserts, and astrocytes were seeded on the opposite side of the Transwell filter of the inserts (Figure 2(c)). The schematic drawing of the triple cell coculture system is shown in Figure 1. The transendothelial electrical resistance (TEER) of different models indicated that the coculture system had an acceptable BBB function (Figure 2(d)). TEM findings demonstrated that BBB appeared normal in rBMEC; meanwhile, tight junctions and desmosomes were close and adjacent (Figure 3).

### 3.1. Effects of TCHi on Cell Survival in NVU Cells after OGD/R

**Figure 4:**
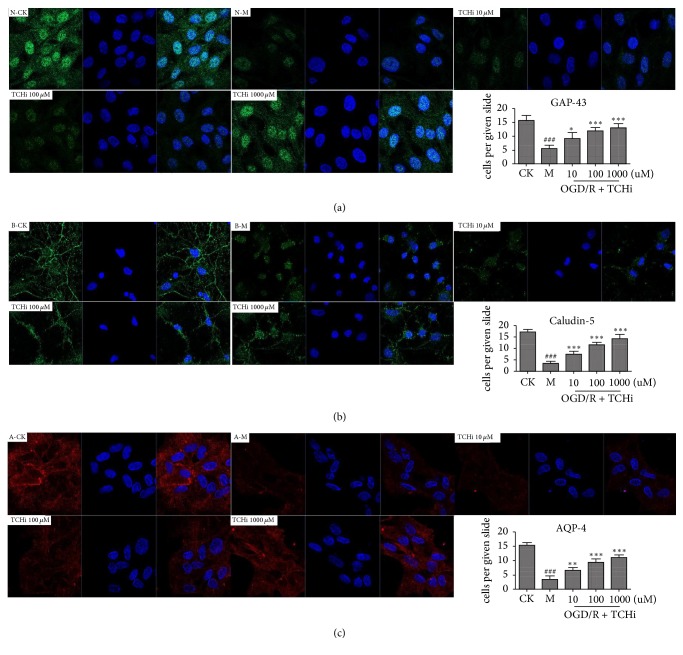
Immunocytochemical analysis demonstrated that TCHi had effects on NVU cells against OGD/R. (a), (b), and (c) reflect neurons, rBMECs, and astrocytes, respectively (GAP-43 is labeled in green in (a), Claudin-5 is labeled in green in (b), AQP-4 is labeled in red in (c), and DAPI is labeled in blue). ^*∗*^*P* < 0.05, ^*∗∗*^*P* < 0.01, and ^*∗∗∗*^*P* < 0.001 relative to Model; ^###^*P* < 0.01 relative to CK.

**Figure 5:**
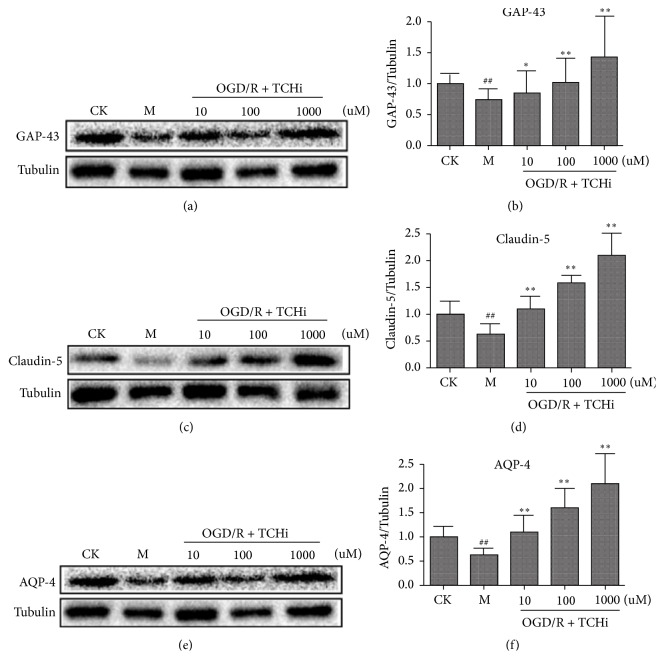
Western blotting showed that TCHi had effects on NVU cells against OGD/R. (a) and (b) are bands of GAP-43 and, on a comparison of densities, indicate that TCHi at concentrations of 10 *μ*M, 100 *μ*M, and 1000 *μ*M maintains neurons after OGD/R. (c) and (d) are bands of Claudin-5 and, on a comparison of densities, indicate that TCHi at concentrations of 10 *μ*M, 100 *μ*M, and 1000 *μ*M maintains rBMECs after OGD/R. (e) and (f) are bands of AQP-4 and, on a comparison of densities, indicate that TCHi at concentrations of 10 *μ*M, 100 *μ*M, and 1000 *μ*M maintains astrocytes after OGD/R.

As shown in Figure 4, neurons, astrocytes, and rBMECs had typical damage manifestations after OGD/R. However, with the treatment of TCHi at doses of 10 *μ*M, 100 *μ*M, and 1000 *μ*M, these manifestations were weakened. Western blotting was analyzed to confirm the effect of TCHi, and the findings were similar to those of immunocytochemical analysis (Figures 4 and 5).

### 3.2. Effects of TCHi on Inflammatory Cytokines in the NVU Model after OGD/R

OGD/R-induced inflammation was inhibited by TCHi in the NVU model. As shown in Table 2, IL-1*β*, IL-6, and TNF-*α* levels decreased significantly in TCHi 10 *μ*M (*P *< 0.01) and TCHi 100 *μ*M (*P* < 0.01). Apart from IL-1*β* and IL-6 (*P* < 0.01), TCHi 1000 *μ*M showed a more obvious effect on TNF-*α* levels (*P* < 0.001).

As well as regulating inflammatory cytokines, cell adhesion molecules, such as vascular cell adhesion molecule 1 (VCAM-1) and intercellular adhesion molecule 1 (ICAM-1), which have the potential to recruit peripheral leukocytes and other cytokines, were upregulated by OGD/R. ELISA results (Table 1) demonstrated that TCHi 10 *μ*M (*P* < 0.05), TCHi 100 *μ*M (*P* < 0.05), and TCHi 1000 *μ*M (*P* < 0.05) ameliorated the increase in the VACM-1 level significantly. The inhibitory effect on ICAM-1 seemed even more apparent in TCHi 1000 *μ*M (*P* < 0.01).

### 3.3. Effects of TCHi on Antiapoptosis in the NVU Model after OGD/R

**Figure 6:**
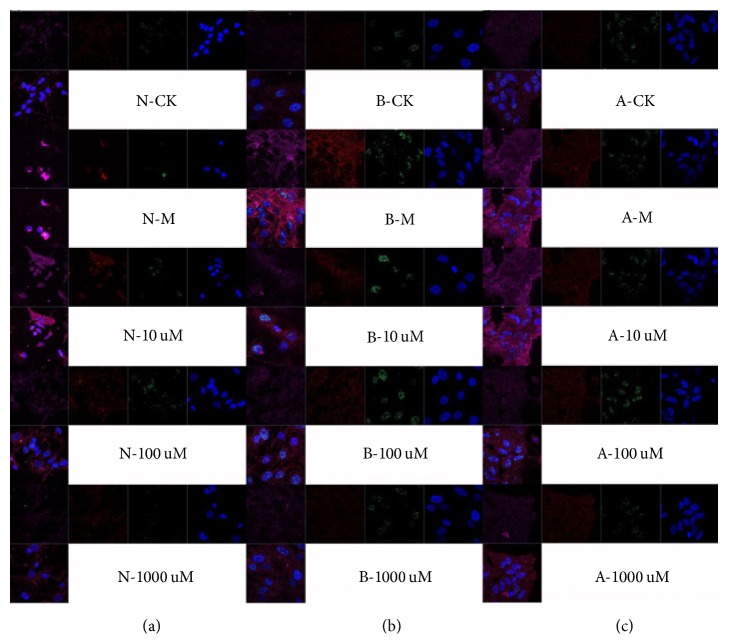
Immunocytochemical analysis demonstrated that TCHi had antiapoptotic effects against OGD/R. (a), (b), and (c) reflect neurons, rBMECs, and astrocytes, respectively (Bax is labeled in orange, p53 is labeled in red, caspase-3 is labeled in green, and DAPI is labeled in blue).

Whether TCHi had effects on apoptosis was further checked. Proapoptotic factors, Bax, p53, and caspase-3, were detected using immunocytochemical analysis.  Figure 6 showed that TCHi at concentrations of 10 *μ*M, 100 *μ*M, and 1000 *μ*M suppressed all these factors.

### 3.4. Effects of TCHi on the Ultrastructure of NVU Cells after OGD/R

**Figure 7:**
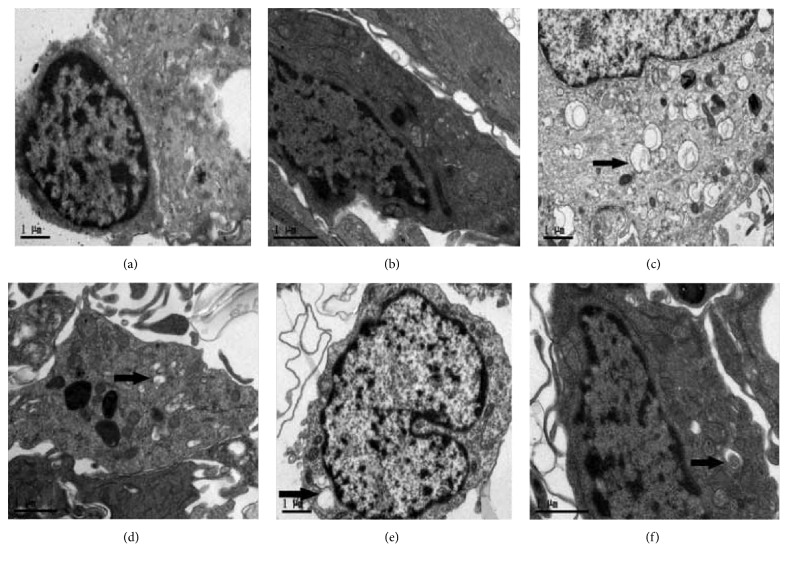
TEM indicated that TCHi alleviated the abnormalities of ultrastructures of neurons and rBMECs. (a) and (b) show that, in normal condition, both neurons and rBMECs exhibit a normal shape and a regular nucleus. (c) and (d) reveal that both neurons and rBMECs show shrinkage of shape, irregular nucleus, diffused distribution of heterochromatin, and amount of autophagosome appearance (black arrow) after OGD/R. (e) and (f) demonstrate that 1000 *μ*M TCHi could reverse the apoptotic appearances of cells.

The ultrastructures of neurons and rBMECs were observed by TEM to confirm the effectiveness of TCHi.  Figure 7 indicated that both neurons and rBMECs showed apoptotic signs after OGD/R. Cells exhibited shrinkage of shape, irregular nuclei, diffused distribution of heterochromatin, autophagosome appearance, and so on. At concentrations of 1000 *μ*M, TCHi reversed all these signs.

## 4. Discussion

Our study demonstrated that an injection of TCH could ameliorate OGD/R, inducing NVU dysfunction. The mechanism of TCH treatment was to suppress inflammation and apoptosis. All these findings in the NVU model implied the underlying therapeutic effect of TCHi against cerebral ischemic strokes, which might explain positive clinical findings for TCHi [9].

Neuroinflammation is a complex inflammatory process in the central nervous system, which is thought to play an important defensive role against various pathogens [15]. However, an aberrant inflammatory response is known to be a type of reperfusion injury after a stroke [16]. Several cytokines and chemokines are released after an ischemic brain injury. The most extensively studied of the proinflammatory cytokines include IL-1*β*, IL-6, and TNF-*α* [16]. This study measured the concentrations of IL-1*β*, IL-6, and TNF-*α* in the OGD/R model and found that TCHi has an inhibitory effect on all these cytokines.

An inflammatory process consists of both the activation of resident cells of the central nervous system and the infiltration of peripheral leukocytes into the ischemic brain tissue [17]. Cell adhesion molecules are involved in the trafficking and recruitment of leukocytes to activate ischemic endothelia in strokes, which could worsen ischemic brain injuries [18]. ICAM-1 and VCAM-1 are reported to be upregulated by the proinflammatory cytokines TNF-*α* and IL-1*β* [19, 20]. The results of the present study revealed that the anti-inflammatory properties of TCHi in an OGD/R-damaged NVU model were due to the reducing levels of ICAM-1 and VCAM-1 as well as cytokines.

Apart from inflammation, apoptosis is another focus in the pathogenesis of brain injuries [17]. The regulation of the apoptosis process is essential to maintain the balance between cell survival and cell death, which is also important in ischemic injuries [21]. Proapoptotic proteins, such as Bax, p53, and caspase-3, were reported to take part in I/R injuries earlier [21]. The current study demonstrated that TCHi could be an effective therapy against apoptosis. It has an impact on the regulation of the apoptosis process via decreasing Bax, p53, and caspase-3 levels in an OGD/R-damaged NVU model.

Not only is BBB the key component of NVU, but also it is the most important structure to maintain cerebral homeostasis and correct neuronal function [22]. BBB integrity in the present study was reflected in TEER, tight junctions, and other ultrastructures of NVUs. TCHi was found to alleviate the BBB breakdown in the OGD/R model. Previous researchers have confirmed the anti-inflammatory and antiapoptotic roles of troxerutin in chronic diseases and diabetic models [23, 24]. The findings of this study revealed that TCHi, a new compound of troxerutin, had a good therapeutic effect in acute attacks such as cerebral I/R injuries.

This study still has limitations. Inflammatory cytokines and cell adhesion molecules were found to be upregulated in NVUs for the* in vitro* OGD/R model; however, the infiltration of peripheral leukocytes and other factors in the central nervous system could not be demonstrated. As a result, further* in vivo* studies should be performed to show whether TCHi had the same effects as in the* in vitro* model.

## 5. Conclusion

TCHi protected the main types of cells of NVUs* in vivo* and* in vitro* depending on anti-inflammation, antiapoptosis, and BBB. The data implies that TCHi is a candidate medicine to treat cerebral ischemic stroke.

## Figures and Tables

**Table 1:** Expression of cytokines in the cell culture medium of NVU (mean ± SD, *N* = 6).

Groups	IL-1*β* (pg/mL)	IL-6 (pg/mL)	TNF-*α* (pg/mL)
CK	280.09 ± 13.36	30.07 ± 5.81	29.56 ± 3.57
Model	568.13±33.36^###^	433.01±20.56^##^	156.49 ± 17.98^###^
TCHi 10 *μ*M	485.57 ± 27.18^*∗∗*^	387.44 ± 30.66^*∗*^	88.43 ± 7.00^*∗∗*^
TCHi 100 *μ*M	446.92 ± 18.57^*∗∗*^	305.90 ± 22.69^*∗∗*^	85.51±2.99^*∗∗*^
TCHi 1000 *μ*M	314.29 ± 20.45^*∗∗*^	289.75 ± 17.49^*∗∗*^	36.06 ± 3.48^*∗∗∗*^

^*∗*^ *P* < 0.05, ^*∗∗*^*P* < 0.01, and ^*∗∗∗*^*P* < 0.001 relative to Model; ^##^*P* < 0.01 and ^###^*P* < 0.01 relative to CK.

**Table 2:** Expression of cell adhesion molecules in the cell culture medium of NVU (mean ± SD, *N* = 6).

Groups	VCAM-1 (pg/mL)	ICAM-1 (pg/mL)
CK	29.03 ± 2.31	21.69 ± 4.40
Model	142.46±6.33^###^	90.31 ± 15.63^###^
TCHi 10 *μ*M	119.88 ± 9.17^*∗*^	73.44 ± 14.61^*∗*^
TCHi 100 *μ*M	104.94 ± 4.13^*∗*^	45.19 ± 22.69^*∗∗*^
TCHi 1000 *μ*M	104.19 ± 4.81^*∗*^	38.97 ± 7.46^*∗∗*^

^*∗*^ *P* < 0.05 and ^*∗∗*^*P* < 0.01 relative to Model; ^###^*P* < 0.001 relative to CK.
